# Optimal balance of benefit versus risk for tafenoquine in the treatment of *Plasmodium vivax* malaria

**DOI:** 10.1186/s12936-024-04924-z

**Published:** 2024-05-13

**Authors:** Raman Sharma, Hema Sharma, Siôn Jones, Isabelle Borghini-Fuhrer, Gonzalo J. Domingo, Rachel A. Gibson, Katie Rolfe, Lionel Tan, Ioana Gabriela Fiţa, Chao Chen, Panayota Bird, Anup Pingle, Stephan Duparc

**Affiliations:** 1 Global Health Medicines R&D, GSK Medicines Research Centre, Gunnels Wood Road, Stevenage, Hertfordshire, SG1 2NY UK; 2 GSK, Brentford, Middlesex UK; 3 Medicines for Malaria Venture, Geneva, Switzerland; 4 PATH, Seattle, WA USA; 5 GSK, Mumbai, India

**Keywords:** *Plasmodium vivax* malaria, Radical cure, Tafenoquine, Benefit–risk

## Abstract

A single 300 mg dose of tafenoquine (an 8-aminoquinoline), in combination with a standard 3-day course of chloroquine, is approved in several countries for the radical cure (prevention of relapse) of *Plasmodium vivax* malaria in patients aged ≥ 16 years. Despite this, questions have arisen on the optimal dose of tafenoquine. Before the availability of tafenoquine, a 3-day course of chloroquine in combination with the 8-aminoquinoline primaquine was the only effective radical cure for vivax malaria. The World Health Organization (WHO)-recommended standard regimen is 14 days of primaquine 0.25 mg/kg/day or 7 days of primaquine 0.5 mg/kg/day in most regions, or 14 days of primaquine 0.5 mg/kg/day in East Asia and Oceania, however the long treatment courses of 7 or 14 days may result in poor adherence and, therefore, low treatment efficacy. A single dose of tafenoquine 300 mg in combination with a 3-day course of chloroquine is an important advancement for the radical cure of vivax malaria in patients without glucose-6-phosphate dehydrogenase (G6PD) deficiency, as the use of a single-dose treatment will improve adherence. Selection of a single 300 mg dose of tafenoquine for the radical cure of *P. vivax* malaria was based on collective efficacy and safety data from 33 studies involving more than 4000 trial participants who received tafenoquine, including over 800 subjects who received the 300 mg single dose. The safety profile of single-dose tafenoquine 300 mg is similar to that of standard-dosage primaquine 0.25 mg/kg/day for 14 days. Both primaquine and tafenoquine can cause acute haemolytic anaemia in individuals with G6PD deficiency; severe haemolysis can lead to anaemia, kidney damage, and, in some cases, death. Therefore, relapse prevention using an 8-aminoquinoline must be balanced with the need to avoid clinical haemolysis associated with G6PD deficiency. To minimize this risk, the WHO recommends G6PD testing for all individuals before the administration of curative doses of 8-aminoquinolines. In this article, the authors review key efficacy and safety data from the pivotal trials of tafenoquine and argue that the currently approved dose represents a favourable benefit–risk profile.

## Background

Since 2018, single-dose tafenoquine 300 mg has been approved in combination with a standard 3-day course of chloroquine, for the radical cure of *Plasmodium vivax* malaria in patients aged ≥ 16 years [1]. Despite approval in several countries, authors of recent articles have questioned the optimal dose of tafenoquine [2–4]. Indeed one such retrospective meta-analysis conducted by Watson et al., suggested that the recommended adult dose of tafenoquine is insufficient for radical cure in all adults and predicted that the risk of relapse would be substantially reduced with an increased adult dose of 450 mg; in response to this, GSK has published a rebuttal, noting that ‘*a tafenoquine dose increase from 300 mg to 450 mg when co-administered with chloroquine is not supported by available fact-based evidence for the radical cure of P. vivax malaria in adults aged* ≥ *16 years*’ [4, 5]. Here, the authors summarize data underlying the favourable benefit–risk profile of single-dose tafenoquine 300 mg.

*Plasmodium vivax* is the most geographically widespread cause of malaria, and is the predominant malarial parasite in South and South-East Asia, Latin America, and North-East Africa, having a substantial global health and economic impact [6, 7]. Following the bite of an infected mosquito, *P. vivax* travels haematogenously to the liver, from where it can exit and cause acute malaria (blood stage). Alternatively, the parasite can remain dormant in the liver as a hypnozoite (liver/hypnozoite stage), reactivating weeks, months, or years later to cause relapses of malaria. Previously considered benign, vivax malaria is responsible for greater morbidity than once understood; it can impact the growth and development of children, as well as cause severe anaemia, pulmonary complications, cerebral malaria, or even death [7–12].

For the effective management of *P. vivax* malaria, eradication of both the blood and liver stages is required (radical cure) [7]. Decades before the approval of tafenoquine, primaquine (an 8-aminoquinoline) in combination with a blood stage treatment (chloroquine or artemisinin-based combination therapy [ACT]), was the only effective regimen targeting hypnozoites and enabling relapse prevention. However, due to its short half-life (6 h), primaquine requires repeated dosing over multiple days for full efficacy, presenting the challenge of ensuring adherence [13–15]. The World Health Organization (WHO)-recommended standard regimen is 14 days primaquine 0.25 mg/kg/day (equivalent to 15 mg/day) in most regions, or 0.5 mg/kg/day in East Asia and Oceania [16]. The WHO recently recommended primaquine 0.5 mg/kg/day for 7 days for uncomplicated *P. vivax* malaria to improve adherence [16].

## Tafenoquine: key findings of clinical efficacy and safety

Tafenoquine is a slowly eliminated 8-aminoquinoline used, in combination with chloroquine, for the radical cure of *P. vivax* malaria [1]. Tafenoquine solves the issue of poor adherence to daily primaquine by providing radical cure in a single dose [17]. The safety profile of single-dose tafenoquine 300 mg is similar to that of standard dose primaquine 0.25 mg/kg/day for 14 days [18]. Primaquine and tafenoquine can cause acute haemolytic anaemia in individuals with glucose-6-phosphate dehydrogenase (G6PD) deficiency; severe haemolysis can lead to anaemia, kidney damage, and even death [19, 20]. G6PD deficiency is a hereditary disorder, with the highest prevalence of G6PD-deficient individuals in malaria-endemic regions [18, 21]. As an X-linked disorder, males are either G6PD-deficient or have normal G6PD activity; females can have deficient, intermediate, or normal G6PD activity; *G6PD* heterozygous females typically have intermediate G6PD activity [7]. Therefore, relapse prevention through adequate 8-aminoquinoline anti-hypnozoite activity must be balanced with the need to avoid clinical haemolysis due to widespread erythrocyte loss associated with G6PD deficiency [20, 22]. To minimize risk, the WHO recommends G6PD testing for all individuals before the administration of curative doses of 8-aminoquinolines, where feasible [8, 23].

The haemolytic potential of tafenoquine was assessed in a phase 1 study of 15 G6PD-deficient heterozygous (G6PD Mahidol variant) females, and the haemolytic risk was dose-dependent (ie, a greater maximum haemoglobin decrease was noted as the tafenoquine dose increased from 100 to 300 mg, and dose-limiting toxicity was evident in 3/3 participants at 300 mg). Mean maximum decreases in haemoglobin were –1.72 g/dL, –1.83 g/dL, and –2.83 g/dL for tafenoquine 100, 200, and 300 mg, respectively [24]. Haemolysis was greatest in participants with the lowest G6PD activity. In addition, the degree of haemolysis associated with single-dose tafenoquine 300 mg was similar to that with primaquine 0.25 mg/kg/day for 14 days [24]. However, concerns have been raised regarding the risk of haemolysis in *G6PD* heterozygous females treated with higher doses of primaquine [25, 26].

To minimize haemolysis risk, G6PD testing should be performed in all patients who may otherwise be eligible for tafenoquine, and tafenoquine should be withheld from any patient with a phenotypic G6PD test result showing enzyme levels < 70% of normal [1, 27]. The 70% threshold for G6PD activity was selected to address the risk of haemolysis in *G6PD* heterozygous females with intermediate G6PD activity.

Although *G6PD* heterozygous females may have apparently normal G6PD activity (> 40%) on qualitative G6PD tests, they can still experience haemolysis with higher primaquine doses [25]. While the long half-life of tafenoquine does not appear to result in a higher risk of haemolysis relative to that of primaquine 0.25 mg/kg/day for 14 days, if haemolysis following tafenoquine or primaquine should progress to more severe acute haemolytic anaemia, the primaquine course can be interrupted, whereas single-dose tafenoquine cannot be stopped once administered [24]. However, studies have shown that for the G6PD A and G6PD Viangchan variants, continued primaquine administration leads to stabilization of haemoglobin levels—termed ‘resistance phase’—which is a state of low-grade haemolysis resulting from higher G6PD activity in the younger red cell population following acute haemolysis [28, 29]. Due to the similarity in haematological parameters, primaquine and tafenoquine may both induce a resistance phase [24].

Approval of single-dose tafenoquine 300 mg for the radical cure of vivax malaria by regulatory agencies around the world, was based on efficacy and safety data from a comprehensive global clinical development programme of 33 studies involving > 4000 trial participants who received tafenoquine at various doses, including over 800 who received the 300 mg single dose [1, 6]. Pivotal data were obtained from three randomized, double-blind studies, in which almost 500 patients with *P. vivax* malaria received tafenoquine 300 mg: DETECTIVE (ClinicalTrials.gov: NCT01376167) part 1 (phase 2b) [30] and part 2 (phase 3) [31]; and GATHER (NCT02216123; phase 3) [18]. In DETECTIVE part 1 (a dose-ranging study), tafenoquine exposure was a significant predictor of efficacy, and there was only a marginal efficacy gain with a 600 versus 300 mg dose: relapse-free efficacy at 6 months was 91.9% (95% CI 80.0–97.0) and 89.2% (95% CI 77.0–95.0), respectively [30]. While the study was designed to have sufficient power to detect a 30% treatment difference between each tafenoquine arm and chloroquine alone, it was not designed to have sufficient power to test for a difference between different tafenoquine doses. Population pharmacokinetics modelling demonstrated that single-dose tafenoquine 300 mg provided systemic exposure greater than the clinically relevant breakpoint obtained in a classification and regression tree analysis (area under the concentration–time curve [AUC] of 56.4 μg⋅h/mL) in approximately 93% of individuals. Consequently, these individuals would have a high probability (85%) of being relapse-free at 6 months [32].

In the phase 3 DETECTIVE part 2 and GATHER studies, the recurrence-free rates at 6 months after single-dose tafenoquine 300 mg in the intention-to-treat populations were 62.4% (95% CI 54.9–69.0) and 72.7% (95% CI 64.8–79.2), respectively; the corresponding recurrence-free rates at 4 months were 73.0% (95% CI 66.0–78.9) and 82.3% (95% CI 74.9–87.7), respectively [18, 31]. Furthermore, a phase 2b, paediatric study (TEACH; NCT02563496) used a pharmacokinetics bridging design to evaluate tafenoquine dose [33]. Participants (aged 2–15 years) received tafenoquine according to body weight to achieve the same median AUC as the 300 mg dose in those aged 16 years or older (children weighing > 10–20 kg received 100 or 150 mg; > 20–35 kg received 200 mg; and > 35 kg received 300 mg). The recurrence-free rate at 4 months was 94.7% (95% CI 84.6–98.3) [33], and the TEACH study supported the approval of tafenoquine for children aged 2–16 years by the Australian Therapeutic Goods Administration in March 2022 and the National Regulatory Agency for Brazil in August 2023 [34, 35].

The efficacy and safety of tafenoquine with anti-malarials other than chloroquine have not been established. Dihydroartemisinin–piperaquine is an artemisinin-based combination used as an alternative to chloroquine for the treatment of *P. vivax* malaria in regions of chloroquine resistance. However, in the INSPECTOR trial (NCT02802501; phase 3) of 150 Indonesian soldiers with normal G6PD activity returning to Java with *P. vivax* malaria after deployment in Papua, single-dose tafenoquine 300 mg plus a 3-day course of dihydroartemisinin–piperaquine produced relapse-free efficacy of only 21% (95% CI 11–34) at 6 months. Corresponding efficacy rates were 11% (95% CI 4–22) for dihydroartemisinin–piperaquine alone and 52% (95% CI 37–65) for dihydroartemisinin–piperaquine plus 15 mg/day primaquine for 14 days [36]. Thus, although there was a statistically significant benefit for tafenoquine plus dihydroartemisinin–piperaquine compared with dihydroartemisinin–piperaquine alone in the radical cure of *P. vivax* malaria, the magnitude of the benefit was not clinically meaningful [36]. A single post-marketing case from a US safety surveillance study also described lack of efficacy for tafenoquine in combination with another artemisinin-based combination, artemether–lumefantrine [37]. The reasons for the lack of efficacy for tafenoquine and ACT are still being investigated. No clinically significant pharmacokinetic drug interactions have been identified between tafenoquine and dihydroartemisinin–piperaquine [38]; however, the possibility of pharmacodynamic interactions with tafenoquine when used with an artemisinin-based combination, instead of chloroquine, needs further exploration. Studies using preclinical models, both in vitro and in vivo, are ongoing, with the aim of exploring possible pharmacodynamic interactions between ACT and 8-aminoquinolines to identify a suitable malaria blood stage treatment (other than chloroquine) that can be used with tafenoquine (Gamo FJ, GSK, personal communication).

A recent modelling study suggests that a higher dose of tafenoquine could result in higher efficacy with little predicted increase in the risk of severe adverse events [4]. Both the incremental benefits but also the risks of a higher dose are difficult to model and predict, as exemplified recently in reported primaquine studies. Doubling the dose of primaquine to 1 mg/kg/day for 7 days versus 0.5 mg/kg/day for 14 days, thus maintaining the same overall dose, resulted in equivalent efficacy [39, 40]. In contrast, both drug-related and drug-unrelated adverse events were significantly higher in the 1 mg/kg/day arm when compared to the 0.5 mg/kg/day dosing [39] and when compared to *Plasmodium falciparum* standard of care treatment with a single low dose (0.25 mg/kg) primaquine administered [41]. A higher risk of haemolysis was also found in females with intermediate G6PD activity [26, 42].

## Conclusion

Real-world efficacy or effectiveness of a drug is defined by multiple factors beyond its efficacy, but relies heavily on: (i) the compliance of healthcare providers prescribing the drug when and as indicated, and (ii) adherence of the patient to the treatment regimen. Radical cure of *P. vivax* with primaquine has suffered from shortcomings for both of these reasons [43, 44]. Tafenoquine solves attrition in efficacy due to poor adherence. As a result, in real-life conditions when tafenoquine is administered in combination with a point-of-care test for G6PD deficiency, the effectiveness of the 300 mg dose regimen is significantly higher than that of multi-day primaquine, in terms of recurrence-free effectiveness at Day 90 and median time to recurrence [45, 46].

Higher doses of tafenoquine are likely to present a higher risk of severe haemolytic events in populations with a significant prevalence of G6PD deficiency, particularly where fragile healthcare systems may result in inappropriate treatment with tafenoquine, in patients with inadequate G6PD activity. The impact of this higher risk as a deterrent on policy for adoption and compliance, therefore reducing overall effectiveness, should be carefully considered.

Radical cure with tafenoquine and primaquine combined with point-of-care testing for G6PD deficiency represents an opportunity in malaria case management in most healthcare settings where *P. vivax* is prevalent. Learning how to safely scale these effective interventions is essential towards understanding future opportunities to optimize them, including higher doses and weight-based dosing among other opportunities that may arise.

The approved tafenoquine 300 mg dose has undergone clinical trials per regulatory requirement, demonstrating a likely optimal balance between efficacy and safety when dosed with 3 days of chloroquine. As haemolytic risk was shown to be dose dependent, with a dose > 300 mg deviation from the approved dose may impact the risk of haemolysis. An alternative dose should not be recommended without evidence in the field of treatment failure, followed by rigorous clinical trials and safety studies in malaria-endemic regions where tafenoquine may be used. Overall, single-dose tafenoquine 300 mg with a 3-day course of chloroquine is an important advancement for the radical cure of *P. vivax* malaria in patients without G6PD deficiency, and the use of a single-dose treatment has the potential to improve adherence.

## Data Availability

Not applicable.

## Abbreviations

ACT: Artemisinin-based combination therapy
AUC: Area under the concentration–time curve
G6PD: Glucose-6-phosphate dehydrogenase
WHO: World Health Organization
